# N6-Methyladenosine in Cell-Fate Determination of BMSCs: From Mechanism to Applications

**DOI:** 10.34133/research.0340

**Published:** 2024-04-25

**Authors:** Qingyu Zhang, Junyou Li, Cheng Wang, Zhizhuo Li, Pan Luo, Fuqiang Gao, Wei Sun

**Affiliations:** ^1^ Department of Orthopedics, Shandong Provincial Hospital affiliated to Shandong First Medical University, Jinan 250021, China.; ^2^ School of Mechanical Engineering, Sungkyunkwan University, Suwon 16419, South Korea.; ^3^ Department of Orthopaedic Surgery, Peking University Third Hospital, Peking University, Beijing 100191, China.; ^4^ State Key Laboratory of Pharmaceutical Biotechnology, Division of Sports Medicine and Adult Reconstructive Surgery, Department of Orthopedic Surgery, Nanjing Drum Tower Hospital, the Affiliated Hospital of Nanjing University Medical School, Nanjing 210008, China.; ^5^ Department of Joint Surgery, Honghui Hospital, Xi’an Jiaotong University, Xi’an 710054, China.; ^6^ Department of Orthopedics, China-Japan Friendship Hospital, Beijing 100029, China.; ^7^ Department of Orthopaedic Surgery of the Perelman School of Medicine, University of Pennsylvania, Philadelphia, PA 19104, USA.

## Abstract

The methylation of adenosine base at the nitrogen-6 position is referred to as “N6-methyladenosine (m^6^A)” and is one of the most prevalent epigenetic modifications in eukaryotic mRNA and noncoding RNA (ncRNA). Various m^6^A complex components known as “writers,” “erasers,” and “readers” are involved in the function of m^6^A. Numerous studies have demonstrated that m^6^A plays a crucial role in facilitating communication between different cell types, hence influencing the progression of diverse physiological and pathological phenomena. In recent years, a multitude of functions and molecular pathways linked to m^6^A have been identified in the osteogenic, adipogenic, and chondrogenic differentiation of bone mesenchymal stem cells (BMSCs). Nevertheless, a comprehensive summary of these findings has yet to be provided. In this review, we primarily examined the m^6^A alteration of transcripts associated with transcription factors (TFs), as well as other crucial genes and pathways that are involved in the differentiation of BMSCs. Meanwhile, the mutual interactive network between m^6^A modification, miRNAs, and lncRNAs was intensively elucidated. In the last section, given the beneficial effect of m^6^A modification in osteogenesis and chondrogenesis of BMSCs, we expounded upon the potential utility of m^6^A-related therapeutic interventions in the identification and management of human musculoskeletal disorders manifesting bone and cartilage destruction, such as osteoporosis, osteomyelitis, osteoarthritis, and bone defect.

## Introduction

Bone has a self-repair ability so that it can renew itself when the nature and extent of the defect are not large, severe, and chronic [1–3]. The reparative process of bone is initiated by bone mesenchymal stem cells (BMSCs) residing in the bone marrow, which are multipotent stem cells with the ability to differentiate into various cell types, including osteoblasts, adipocytes, chondrocytes, and fibroblasts [1,2,4]. Data analysis revealed two distinct phases of the BMSC differentiation process, namely, lineage commitment (from MSCs to lineage-specific progenitors) and maturation (from progenitors to specific cell types) [2]. The dysregulation of adipose-osteogenic balance could result in the formation of excessive fat and compromised bone structure, contributing to the pathogenesis of multiple musculoskeletal conditions [5,6]. The differentiation of BMSCs is regulated by the activation of specific intracellular transcription factors (TFs), signaling pathways, and noncoding RNAs (ncRNAs; e.g., miRNA, lncRNA, and circRNA) [2,7]. In addition, extracellular elements such as hypoxia and mechanical stimulation are also involved in these vital processes [8].

N6-methyladenosine (m^6^A) refers to the methylation at the nitrogen-6 position of adenosine, which normally uses S-adenosylmethionine (SAM) as the methyl donor, and ranks as one of the most abundant and conserved epigenetic modifications of messenger RNA (mRNA) and ncRNA in eukaryotes [9,10]. m^6^A modification is reversible, achieved by proteins known as m^6^A “writers,” “erasers,” and “readers.” Recent studies have shown that aberrant m^6^A levels caused by methyltransferase-like 3 (METTL3) are involved in the development and progression of numerous malignancies [e.g., lung cancer and acute myeloid leukemia (AML)], inflammatory diseases, metabolic disorders, and cardiovascular diseases [11–13]. However, the role of m^6^A modification in bone homeostasis is little known. Increasing evidence has suggested that m^6^A modification is critical for the differentiation, proliferation, apoptosis, and necrosis of BMSCs, but these findings have not been comprehensively summarized. In this article, we will briefly elaborate on the biological function and clinical value of m^6^A modification in the differentiation of BMSCs, which may provide possible targets for diagnosing and treating human musculoskeletal diseases such as osteoporosis, osteoarthritis, and osteomyelitis.

## Brief Overview of m^6^A Modification

The m^6^A methyltransferase complex (MTC; writers) catalyzing the formation of most mRNA m^6^A consists of a METTL3/METTL14 heterodimer core and other binding partners represented by Wilms tumor 1-associating protein (WTAP) [11,12]. Among this complex, METTL3 is of the most interest from a research standpoint because it is the sole catalytically active subunit of MTC implicated in RNA biogenesis, translation, and degradation, serving as a core protein for m^6^A modification [11,12,14]. Readers are m^6^A methyl-binding proteins and mainly include YTHDC1/2, YTHDF1/2/3, and IGF2BP1/2/3 [14,15]. The m^6^A erasers refer to the demethylases like FTO and ALKBH5 [14,15].

**Fig. 1.**
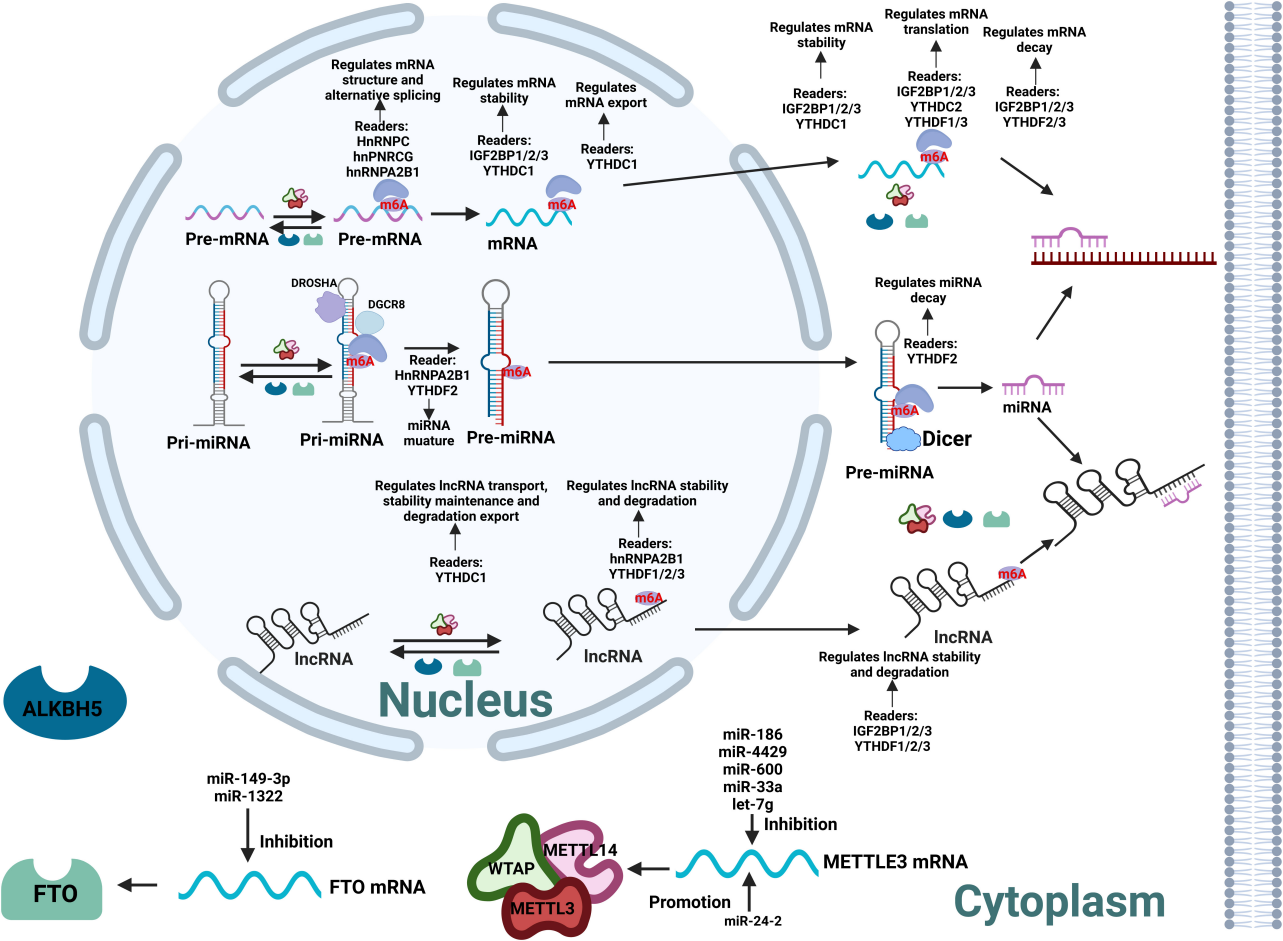
Interaction between m^6^A modification, miRNA, lncRNA, and mRNA during BMSC differentiation. m^6^A modification is synergistically catalyzed by methyltransferases (writers), demethylases (erasers), and methyl-binding proteins (readers). Readers including YTH family, HNRNP family, and IGFBP1/2/3 paly vital roles in determining the fate of methylated RNAs.

In addition to directly regulating the expression of mRNAs, m^6^A modification of ncRNAs, especially lncRNAs and miRNAs, has garnered growing interest in recent years, although the number of related studies is much lower (Fig. 1) [14,16]. NcRNAs and m^6^A may cooperate or compete to jointly regulate target mRNAs [14,16]. Moreover, previous studies have suggested that ncRNAs are the main sites of RNA epigenetic modification [17].

### m^6^A modification regulates the metabolism of mRNA

METTL3 located in the nucleus affects the maturation and transportation of mRNAs [18]. The widespread m^6^A deposition in pre-mRNA was associated with the mRNA alternative splicing, producing diverse mature mRNA sequences and substantial cellular complexity [19]. Stimulation effects of m^6^A modification on mRNA transportation from the nucleus to the cytoplasm were also unraveled, which were vital for active translations in eukaryotes [19,20]. In the METTL3-enriched cytoplasm, m^6^A modification plays an intricate effect on mRNA, mediating mRNA translation efficiency, stability, or degradation depending on the corresponding m^6^A reader (e.g., YTHDC1/2, YTHDF1/2/3, and IGF2BP1/2/3) [19–22]. Apart from its eminent methyltransferase activity, it has been reported that METTL3 could facilitate the translation initiation of transcripts harboring the m^6^A-modified 3′-UTR (untranslated region) without the help of m^6^A reader proteins [15,23].

### Mutual regulatory effects between m^6^A modification and ncRNAs

m^6^A modification could enhance or decrease the transcript stability of modified lncRNA, alter the subcellular distribution, mediate gene transcription repression, change the lncRNA structures, and affect the interaction with associated proteins [24,25]. The specific methyl-binding proteins that impact the transcript stability of lncRNA are also summarized in Fig. 1. At the same time, mutual regulation exists between lncRNA and METTL3. Shen et al. [26] demonstrated that aspartyl-tRNA synthetase 1 antisense 1 (DARS-AS1), an oncogenic lncRNA, could facilitate the translation of DARS by enlisting METTL3 and METTL14 in cervical cancer.

In mammalian cells, m^6^A modification of pri-miRNAs facilitates recognition by DGCR8 and enhances miRNA maturation in a global and non-cell type-specific manner [27]. m^6^A modification could also promote the synthesis of mature miRNAs by accelerating the splicing of pre-miRNAs by Dicer [28]. METTL3 depletion contributes critically to the global reduction of mature miRNAs and concomitant overaccumulation of unprocessed pri-miRNAs, hinting that the m^6^A mark acts as a key posttranscriptional modification that boosts the initiation of miRNA biogenesis [14]. Simultaneously, certain miRNAs such as miR-186, miR-4429, miR-600, miR-33a, and let-7g can directly target mRNAs of METTL3 and result in translation inhibition [19] [15]. It was also suggested that miR-24-2 might induce METTL3 transcription, although the comprehensive and detailed mechanism was poorly understood [29].

## m^6^A Modification and Osteogenesis of BMSCs

Expression of METTL3 and m^6^A content in total RNA was robustly up-regulated in BMSCs undergoing osteogenic differentiation [30]. METTL3 loss of function in BMSCs lowers the mRNA level of bone formation-related genes, such as Runt-related transcription factor 2 (RUNX2), osteocalcin (OCN), osteopontin (OPN), and alkaline phosphatase (ALP), hampering osteogenic differentiation and the formation of mineralized nodules [31–33]. In contrast, adenovirus-mediated overexpression of METTL3 produced the opposite effects. However, the expression and influence of FTO and ALKBH5 on osteogenesis of BMSCs are debatable [34–37]. Down-regulation or up-regulation of this m^6^A demethylase can both disrupt osteogenic differentiation.

### m^6^A modification of TFs during osteogenesis (Table 1 and Fig. 2)

**Table 1.** Modified genes by m^6^A modification in osteogenesis, adipogenesis, and chondrogenesis of BMSCs

Differentiation process	Target/pathway	Key molecule of writer	Reader	Regulation	Function
Osteogenesis	Runx2 mRNA [30]	METTL3	YTHDF1, IGF2BP1 [33]	Promotes RNA stability and translation	Promotor
Osterix mRNA [46]	METTL3	NR	Up-regulation	Promotor
Smad7 mRNA and Smurf1 mRNA [40]	METTL3	YTHDF2	Promotes RNA degradation	Promotor
BMP2 mRNA [44]	METTL3	YTHDF2	Promotes RNA degradation	Inhibitor
Clip3 mRNA [50]	METTL3	NR	Promotes RNA degradation	Promotor
VEGFA/VEGFA-164/VEGFA-188 mRNA [46]	METTL3	NR	Up-regulation	Promotor
pth1r mRNA [49]	METTL3	NR	Promotes RNA translation	Promotor
PTPN6 mRNA [51]	METTL14	NR	Up-regulation	Promotor
TCF1 mRNA [52]	METTL14	NR	Up-regulation	Promotor
Beclin-1 [53]	METTL14	IGF2BPs	Promotes RNA stability and translation	Promotor
SMAD1 [54]	METTL14	IGF2BP1	Promotes RNA stability and translation	Promotor
Adipogenesis	JAK1 mRNA/ JAK1-STAT5-C/EBPβ pathway [109]	METTL3	YTHDF2	Promotes RNA decay	Inhibitor
CCND1 mRNA [115]	METTL3	YTHDF2	Promotes RNA decay	Inhibitor
AKT1 mRNA [106]	METTL3	YTHDF2	Promotes RNA decay	Inhibitor
Chondrogenesis	SOX9 mRNA [123]	METTL3	YTHDF2	Promotes RNA translation	Promotor
MMP3 [124]	METTL3	NR	Up-regulation	Promotor
MMP13 [124]	METTL3	NR	Up-regulation	Promotor
GATA [124]	METTL3	NR	Up-regulation	Promotor

NR, not reported

**Fig. 2.**
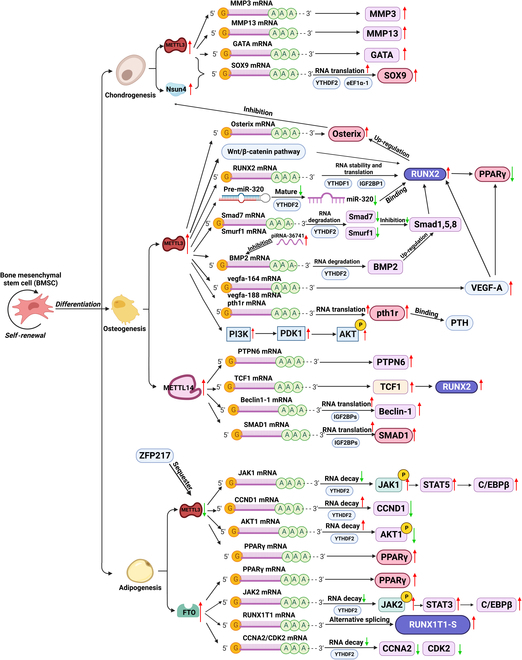
Molecular mechanisms underlying regulation of m^6^A modification and demethylation of target genes in osteogenesis, adipogenesis, and chondrogenesis of BMSCs. The m^6^A marks of the target transcripts recruit m^6^A reader proteins to determine RNA fate.

RUNX2 and Osterix are two essential TFs for the osteoblastic differentiation of BMSCs and skeletal morphogenesis, localized within both the nucleus and cytosol [38]. Most signaling pathways (e.g., BMP2/Smad pathway and Wnt/β-catenin pathway) investigated during osteogenesis so far are targeted at RUNX2 [39,40]. RUNX2 is enhanced by core-binding factor β (Cbfβ) and therefore activates OCN, OPN, ALP, and bone sialoprotein [38,41]. Up-regulated RUNX2 in BMSCs elevates their lineage commitment into osteoblasts and impedes their differentiation potential into adipocytes by disturbing the peroxisome proliferator-activated receptor γ (PPARγ) pathway (Fig. S1) [42]. RUNX2 is decreased during maturated osteoblasts, while Osterix is obligatory for the maturation process [43].

RUNX2 is affected by m^6^A RNA methylation mainly through the dual signaling cascades of osteogenic pathways. On the one hand, METTL3 directly up-regulates m^6^A methylation of RUNX2, increasing RUNX2 mRNA stability and translation by the recognition of YTHDF1 and IGF2BP1 [33,36]; on the other hand, METTL3 promotes m^6^A methylation of pre-miR-320 and inhibits the maturation of miR-320 by YTHDF2, which elevates the expression of associated bone fide target genes for miR-320 family including RUNX2 [30]. The BMP2/Smad pathway, which serves as a positive regulator of RUNX2 expression as well as osteoblastic differentiation, is also regulated by m^6^A modification [39,40]. METTL3 decreased the mRNA maturation and stability of negative regulatory proteins of Smad signaling, *Smad* and *Smurf1* [40]. This inhibitory effect might be reduced by the silence of YTHDF2 [44]. m^6^A modification of BMP2 transcript accelerates mRNA degradation mediated by YTHDF2 [44]. Nevertheless, the osteogenesis up-regulated piR-36741 could combine with PIWIL4 to create a complex and competitively bind to METTL3 with BMP2 mRNA, decreasing METTL3’s m6A activity without altering its level and increasing BMP2 expression [44]. Vascular endothelial growth factor (VEGF) controls the differentiation of BMSCs by regulating RUNX2 and PPARγ as well as through a reciprocal interaction with nuclear envelope proteins lamin A/C [45]. METTL3 could influence the alternative splicing of VEGFA mRNA, increasing the expression level of VEGFA as well as its splice variants, vegfa-164 and vegfa-188 [46].

Osterix/Sp7 is a member of the Sp1 TF family of C_2_H_2_-type zinc finger TFs, which functions as a downstream of RUNX2 [47]. Osterix, along with RUNX2 and Dlx5, drives the differentiation of mesenchymal precursor cells into osteoblasts and eventually osteocytes, and inhibits chondrocyte differentiation, maintaining the balance between osteogenesis and chondrogenesis [48]. The m^6^A methylation of Osterix could also improve the expression of this key osteogenic TF [46]. It is noteworthy that the pre-mRNA of Osterix harbors 56 potential m^6^A modification sites according to a sequence-based m^6^A modification site predictor (http://www.cuilab.cn/sramp). By which mechanism m^6^A modification affects the transcription and expression of Osterix should be further investigated.

### m^6^A modification and affected key genes and signaling pathway during osteogenesis (Table 1 and Fig. 2)

Parathyroid hormone receptor-1 (Pth1r), a vital modulator of lineage commitment in BMSCs and osteoblast precursors, shows a highly concentrated and distinctive m^6^A peak adjacent to its translation stop codon [49]. Wu *et al*. [49] found that the translation efficiency of pth1r mRNA was decreased and the parathyroid hormone (PTH)-induced osteogenic effect was hindered after the knockout of METTL3, which confirmed that m^6^A modification regulates the lineage allocation of MSCs partially by the PTH/Pth1r signaling pathway. In rat BMSCs isolated from osteoporosis models, overexpressing METTL3 restored the osteogenic ability by activating the Wnt/β-catenin signaling pathway and subsequently increased the expression of β-catenin, RUNX2, OPN, P-Gsk-3β, and Lef1 [31]. Mass of genes linked to osteogenic differentiation and bone mineralization were impacted by METTL3 knockdown, with the phosphatidylinositol 3-kinase/AKT (PI3K-Akt) signaling pathway appearing to be among the most abundant pathways [46]. During osteogenesis, an incremental m^6^A level located in the 3′-UTR of the CAP-Gly domain-containing linker protein 3 (Clip3) mRNA was detected, which leads to accelerated degradation of mRNA and down-regulated Clip3 expression [50].

Although METTL14 only engages in the complex stabilization and RNA recruitment of MTC, it could promote osteogenesis of BMSCs in physiological and pathological conditions by stimulating m^6^A modification of multiple mRNAs including PTPN6 [51], TCF1 [52], Beclin-1 [53], and SMAD1 [54].

### m^6^A modification and ncRNAs in osteogenesis of BMSCs (Tables 2 and 3 and Fig. 3)

**Table 2.** Summary of miRNAs mediated by m^6^A modification in osteogenic differentiation

Modulated miRNA	Key molecule of writer	Reader	The function of m^6^A modification	Effect on osteogenesis and associated targets	
Promotion/inhibition				Target genes/pathways
pre-miR-320 [30]	METTL3	YTHDF2	miRNA decay	Promotion	RUNX2 [30]
pri-miR-21 [55]	METTL3	HNRNPA2B1	miR-21-5p mature	Promotion	PTEN/PI3K/Akt/HIF-1α pathway [57]
Smad7-Smad1/5/8-Runx2 pathway [56]				
pri-miR-873 [60]	METTL14	NR	miRNA mature	Promotion	HDAC1 [60]
pri-miR-181a/c [59]	WTAP	YTHDC1	miRNA mature	Promotion	SFRP1 [59]
pri-miR-29b-3p [58]	WTAP	NR	miRNA mature	Promotion	HDAC4 [58]
pri-miR-221/222 [81]	METTL3	HNRNPA2B1	miRNA mature	Inhibition	RUNX2 [82]
Smad3 [84]				
IGF-1/ERK pathway [83]				
pri-miR-143-3p [66]	METTL3	HNRNPA2B1	miRNA mature	Inhibition	KLF5 [61]
STMN1 [65]				
IGFBP5 [63]				
NFIC [64]				
ARL6 [62]				
pri-miR-25 [67–69]	METTL3	NKAP[69]	miR-25-3p mature	Inhibition	Smad5 [71]
ITGB3 [70]				
pri-miR-146a-5p [72,73]	METTL3	HNRNPA2B1	pri-miR-146a-5p mature	Inhibition	Sirt1 [74]
pri-miR-30b-5p [75]	METTL3	HNRNPA2B1	miR-30b-5p mature	Inhibition	BCL6 [76]
pri-miR-93-5p [77]	METTL3	HNRNPA2B1	miR-93-5p mature	Inhibition	BMP-2 [78]
pri-miR-375-3p[79]	METTL3	HNRNPA2B1	miR-375-3p mature	Inhibition	LRP5, β-catenin [80]

NR, not reported

**Table 3.** Summary of lncRNAs mediated by m^6^A modification in osteogenic differentiation

Modulated lncRNA	Key molecule of writer	Reader	The function of m^6^A modification	Effect on osteogenesis and associated targets	
Promotion/inhibition				Target genes/pathways
LINC00657 [105]	METTL3	NR	Up-regulation	Promotion	miR-144-3p/BMPR1B axis [105]
MALAT1 [86]	METTL3	NR	Up-regulation	Promotion	miR-143/OSX [86]
miR-204/Smad4 [85]				
NEAT1 [88]	METTL3	NR	Up-regulation	Promotion	miR-29b-3p/BMP1 [89]
DANCR [133]	METTL3	NR	Up-regulation	Inhibition	miR-1301-3p/PROX1 [129]
EZH2[131]				
Wnt/β-catenin pathway [128,130]				
p38 MAPK pathway [132]				
H19 [102]	METTL3	NR	Up-regulation	Promotion	miR-541-3p/Wnt/β-catenin [91]
miR-625-5p/Wnt/β-catenin [90],				
miR-675/APC/Wnt/β-catenin [92]				
miR-141/Wnt/β-catenin [93],				
miR-22/Wnt/β-catenin [93]				
miR-675/TGF-β1/Smad3/HDAC [104]				
miR-214-5p/BMP2 [94]				
miR-532-3p/SIRT1 [95]				
miR-188/LCoR [96]				
miR-149/SDF-1 [97]				
miR-140-5p/SATB2 [98]				
miR138/FAK [99]				
miR-19b-3p [100]				
p53 [101]				

NR, not reported

**Fig. 3.**
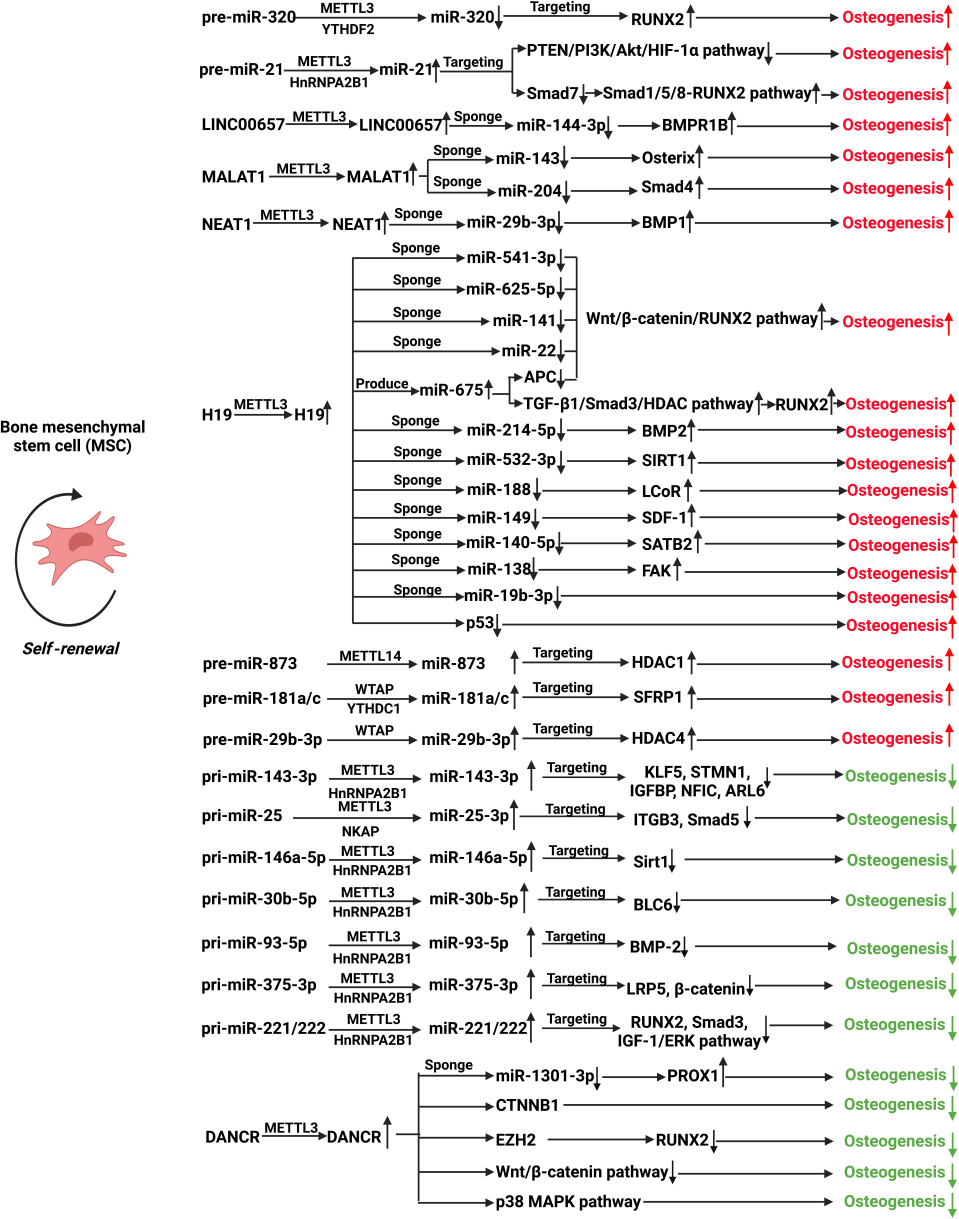
Different ncRNAs and associated pathways modulated by m^6^A modification in regulating osteogenesis of BMSCs.

In addition to abovementioned miR-320, further studies uncovered that METTL3 can methylate pri-miR-21 and facilitate the maturation of miR-21 [55], which potentiates the osteogenesis of BMSCs by activating the Smad1/5/8-RUNX2 pathway [56] and lowering the amount of hypoxia-inducible factor-1α (HIF-1α) [57]. WTAP, another critical component of m^6^A writers, also acts as a promoter of osteogenesis by encouraging mature miR-29b-3p [58] and miR-181a/c [59]. Similar phenomenon was observed between METTL14 and miR-873 [60]. However, some anti-osteogenic miRNAs, such as miR-143-3p [61–66], miR-25-3p [67–71], miR-146a-5p [72–74], miR-30b-5p [75,76], miR-93-5p [77,78], miR-375-3p [79,80], and miR-221/222 [81], were also modulated by m^6^A modification, which stimulates the miRNA mature. These miRNAs could negatively regulate the osteogenic process by binding mRNAs of pro-osteogenic genes. As an illustration, miR-25-3p specifically targets Smad5 [71] and ITGB3 [70], while miR-221/222 could block RUNX2, Smad3, as well as the insulin-like growth factor 1 (IGF-1)/extracellular signal-regulated kinase (ERK) pathway [82–84].

Some well-recognized lncRNAs enhancing osteogenesis, such as MALAT1 [85–87], NEAT1 [88,89], and H19 [90–102], could also be modulated by m^6^A modification to increase the stability [103]. Among them, H19 could sponge multiple miRNAs with negative regulatory effects on the Wnt/β-catenin/RUNX2 pathway [90–94], and produce miR-675 to facilitate the production of RUNX2 [104]. MALAT1 could sponge miR-143 [86] and miR-204 [85] to boost the expression of Osterix and Smad4, respectively. Besides, the osteogenic ability of METTL3 on human BMSCs was partially realized through the m^6^A methylation of LINC00657 and the inhibition of downstream miR-144-3p/BMRPB1 axis [105].

### m^6^A modification in macrophages and osteogenesis of BMSCs

METTL3 in other types of cells in the bone microenvironment could also influence the osteogenesis of BMSCs. Overexpression of METTL3 was identified in the pro-inflammatory type of blood-derived and bone marrow-derived M1 macrophages as compared with non-activated macrophages (M0) [106]. METTL3 overexpression promoted the expression and m^6^A modification of DUSP14, HDAC5, and Nfam1, which has been reported to slow down the onset of osteoporosis [107].

## m^6^A Modification and Adipogenesis of BMSCs

Numerous investigations have revealed that fat induction factors suppress osteogenesis; rather, osteogenic factors restrain adipogenesis [108]. A negative correlation exists between METTL3 expression and BMSC adipogenesis. METTL3 overexpression reduced lipid droplet formation and dramatically suppressed adipogenic markers PPARγ, C/EBPα (CCAAT/enhancer binding protein α), and FABP4 [109]. FTO is a well-known gene linked to obesity that has the ability to control adipogenesis by m^6^A demethylation [110].

### m^6^A modification of TFs during adipogenesis (Table 1 and Fig. 2)

PPAR*γ* and C/EBPs (C/EBP*α*, C/EBP*β*, and C/EBP*δ*) are critical TFs involved in the adipogenic differentiation of BMSCs [111]. After adipogenic differentiation is induced, C/EBP*β* and C/EBP*δ* are swiftly (within 4 h) elevated and subsequently activate C/EBPα and PPARγ [112]. The expression of adipogenic genes that underlie terminally differentiated adipocyte phenotype is coordinated by C/EBPα and PPARγ combined [111,112]. While PPARγ and C/EBPα expression remains high throughout the adipogenic process and the adipocytes’ lifetime, C/EBPβ is down-regulated in the later stages of differentiation [111,112].

In particular, METTL3 blocked the adipogenic differentiation of pBMSCs by interfering with the Janus kinase 1 (JAK1)–signal transducer and activator of transcription 5 (STAT5)–C/EBPβ pathway in a way dependent on m^6^A and YTHDF2 [109]. Deletion of METTL3 significantly decreased mRNA m^6^A levels of JAK1 to augment its stability [109]. By controlling its phosphorylation, JAK1 may bind to the promoter of C/EBPβ and activate the signal transducer and activator of STAT5, which may trigger a modified adipogenic pathway [109]. Meanwhile, adipogenesis is inhibited by deletion of m^6^A demethylase FTO via JAK2-STAT3-C/EBPβ signaling [113]. Increased METTL3 in BMSCs reduced PPARγ expression, while METTL3 knockdown had the opposite impact [106]. Conversely, FTO bonded to and demethylated the PPARγ mRNA, which increased the mRNA's expression [114]. PPAR*γ* and C/EBPs all carry potential m^6^A modification sites, but whether direct m^6^A modification of these TFs influences the adipogenic differentiation of BMSCs merits further instigation.

ZFP217 is a TF belonging to the Krüppel-type zinc finger protein family and has been proven to take involvement in adipogenesis [115]. The last evidence showed that the adipogenesis induced by ZFP217 knockdown was caused by CCND1, which was mediated by METTL3 and YTHDF2 in an m^6^A-dependent manner [115]. Runt-related transcription factor 1 translocation partner 1 (RUNX1T1) is another novel adipogenic regulatory factor [116]. By modulating the amounts of m^6^A around splice sites, FTO regulates the exonic splicing of RUNX1T1, controlling RUNX1T1-S isoform expression and therefore modulating adipogenesis [117].

### m^6^A modification and affected key genes and signaling pathway during adipogenesis (Table 1 and Fig. 2)

Patients with AML have elevated AKT1-mRNA and protein expression due to loss of METTL3, which mediates m^6^A modification of AKT1-mRNA [106]. This increases the likelihood that MSCs will develop into adipocytes, altering the microenvironments of the bone marrow. The aggregates of adipocytes in bone marrow contribute to chemoresistance in AML. This is in line with former findings that METTL3 speeds up the progression of hematological malignancies [13]. Furthermore, FTO has an impact on the cell cycle. YTHDF2 separates and destabilizes m^6^A-modified mRNA of two cell cycle regulators, CCNA2 and CDK2 [118]. FTO can demethylate and boost the expression of CCNA2 and CDK2, which in turn shortens the cell cycle and increases adipogenesis of BMSCs.

### m^6^A modification and key ncRNAs in adipogenesis of BMSCs (Table 4 and Fig. 4)

**Table 4.** Summary of miRNAs mediated by m^6^A modification in adipogenic differentiation

Modulated miRNA	Key molecule of writer	Reader	The function of m^6^A modification	Effects on adipogenesis and associated targets	
Promotion/inhibition				Promotion/inhibition
miR-221/222 [81]	METTL3	HNRNPA2B1	miRNA mature	Promotion	Ddit4 [119]
pri-miR-25 [67–69]	METTL3	NKAP [69]	miR-25-3p mature	Inhibition	KLF4, C/EBPα [28]

**Fig. 4.**
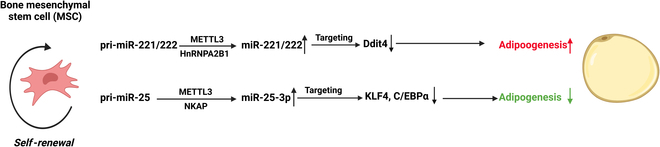
Different ncRNAs and associated pathways modulated by m^6^A modification in regulating adipogenesis of BMSCs.

As mentioned before, the m^6^A modulation accelerates the maturation of pri-miR-221/222 and pri-miR-25-3p. Besides inhibiting osteogenesis-related genes, miR-221/222 could directly boost the adipogenesis processes by targeting Ddit4 [119]. Meanwhile, m^6^A modification accelerated the mature process of miR-25-3p, which acts as a molecular sponge for KLF4 and C/EBPα and could inhibit adipogenesis [120]. Two miRNAs, namely, miR-149-3p and miR-1322, could inhibit adipogenesis of BMSCs by targeting FTO [121,122].

## m^6^A Modification and Chondrogenesis of BMSCs

During chondrogenesis of BMSCs, the protein and mRNA levels of METTL3 were substantially elevated, which recruits more Nsun4 to form a complex [123]. Similar results were obtained in the synovium-derived mesenchymal stem cells (SMSCs) [124]. In chondrogenic differentiation of SMSCs, the m^6^A levels were markedly increased and only protein level METTL3 was most obviously raised in comparison with other m^6^A-related genes [124]. Knockdown of METTL3 suppressed the chondrogenesis of BMSCs and SMSCs [123,124].

### m^6^A modification of TF during chondrogenesis (Table 1 and Fig. 2)

SRY-related high-mobility group box 9 (Sox9) is a critical TF that mediates chondrocyte lineage commitment of BMSCs, benefiting chondrocyte survival by transcriptionally activating the expression of chondrocyte-specific components and regulatory factors, such as collagen type II, type IX, and type XI and aggrecan [125]. The expression of Sox9 was also modulated in the level of epigenetic modification. Nsun4 mediates the m^5^C alteration in the 3′-UTR of Sox9 mRNA, while METTL3 mediates m^6^A modification [123]. Together, these modifications co-regulated the translational reprogramming by creating a complex with YTHDF2 and eEF1α-1. In vivo, BMSCs overexpressing METTL3 and Nsun4 can help repair cartilage defects caused by drilling [123]. Knockdown of METTL3 dramatically reduced the expression of SOX9 [124].

### m^6^A modification and key genes and signaling pathway during chondrogenesis (Table 1 and Fig. 2)

According to the Kyoto Encyclopedia of Genes and Genomes (KEGG) pathway enrichment analysis, the reduction of METTL3 decreased the protein level of MMP3, MMP13, and GATA, which are involved in signaling pathways regulating the glycosaminoglycan biosynthesis–chondroitin sulfate/dermatan sulfate and extracellular matrix (ECM)–receptor interaction [124].

### m^6^A modification and key ncRNAs in chondrogenesis of BMSCs (Table 5 and Fig. 5)

**Table 5.** Summary of ncRNAs mediated by m^6^A modification in chondrogenesis differentiation

Modulated miRNA	Key molecule of writer	Reader	The function of m^6^A modification	Effects on chondrogenesis and associated targets	
Promotion/inhibition				Target genes/pathways
DANCR [133]	METTL3	NR	Up-regulation	Promotion	Smad3/STAT3 [135]
miR-1305/Smad 4 [134]				
MEG3 [136]	METTL3	NR	Up-regulation	Promotion	miR-129-5p/RUNX1 [136]
miR-221 [81]	METTL3	HNRNPA2B1	miRNA mature	Inhibition	TRPS1/Mdm2 [126]
miR-143-3p [16]	METTL3	HNRNPA2B1	miRNA mature	Inhibition	BMPR2 [127]

NR, not reported

**Fig. 5.**
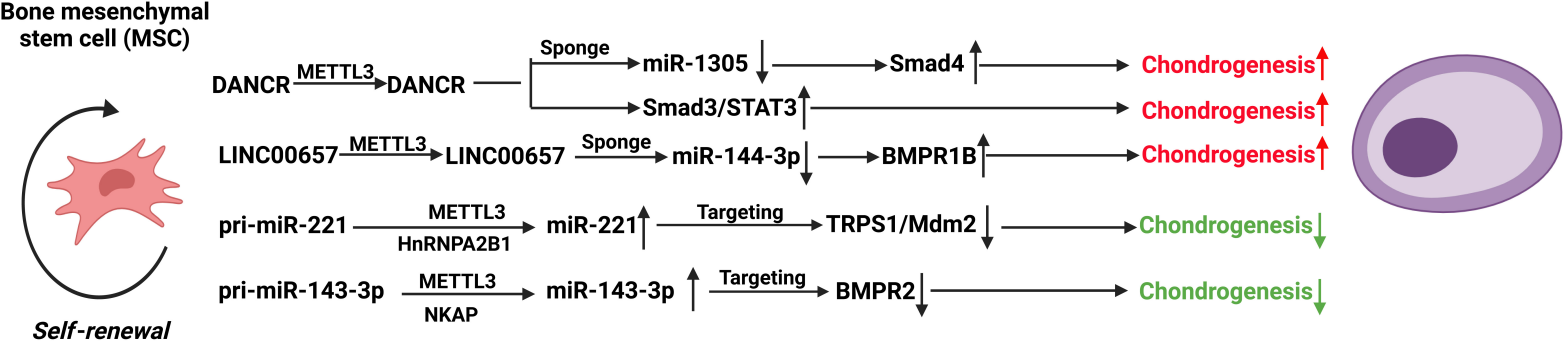
Different ncRNAs and associated pathways modulated by m^6^A modification in regulating chondrogenesis of BMSCs.

MiR-221 is an anti-chondrogenic miRNAs in human mesenchymal stem cells by targeting TRPS1/Mdm2, and silencing this miRNA could contribute to cartilage repair in vivo [126]. Meanwhile, by targeting BMPR2, miR-143-3p also negatively regulates the chondrogenic differentiation of BMSCs [127]. Pri-miRNAs of these two anti-chondrogenic miRNAs could be modulated by METTL3 to encourage the formation of mature miRNAs. DANCR, which could restrain osteogenesis of BMSCs [128–133], is a positive regulator of chondrogenic differentiation by targeting miR-1305, myc, Smad3, STAT3, and Smad4 [134,135]. METTL3 could increase DANCR stability via m^6^A modification. MEG3 can interact with the miR-129-5p/RUNX1 axis to help BMSCs differentiate into chondrocytes, but the stability of MEG3 RNA was compromised after m^6^A modification by METTL3 [136].

## Activators and Inhibitors Targeting m^6^A Modification

The development of chemical tools targeting m^6^A modification (e.g., METTL3 activators/ inhibitors and FTO inhibitors) has aroused considerable interest in treating multiple disorders in the last decade [137,138].

**Fig. 6.**
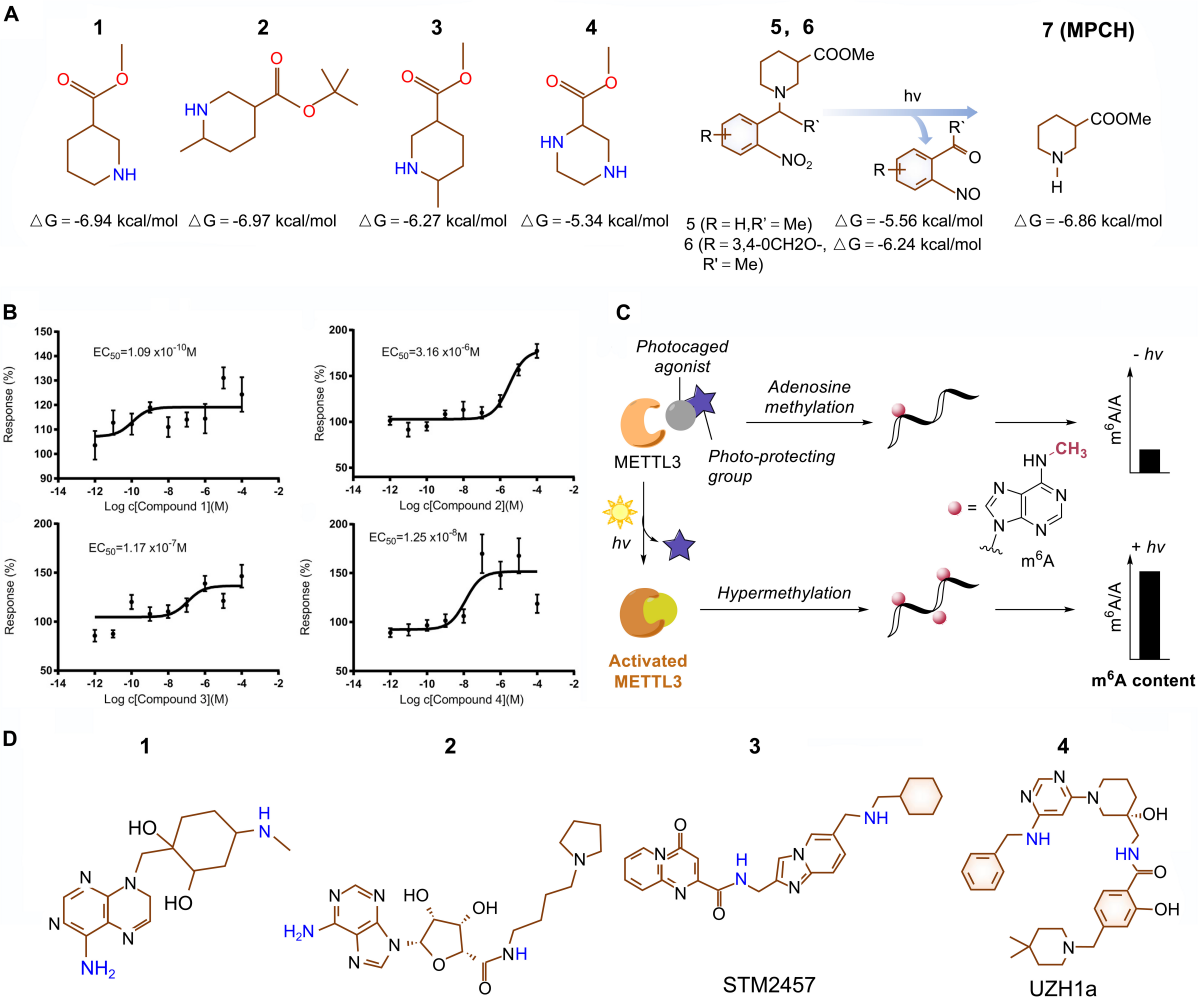
The lead compounds targeting METTL3. (A) Chemical structure of activators targeting METTL3. (B) Influence of activators 1 to 4 of the METTL3–METTL14–WTAP complex on the substrate RNA methylation. Reproduced with permission from [139]. Copyright (2019) the Author(s). (C) Concept of a light-triggered RNA methylation of adenosine promoted by the methyltransferase METTL3. Reproduced with permission from [140]. Copyright (2021) Wiley-VCH GmbH. (D) Chemical structure of inhibitors targeting METTL3.

The potency of four small compounds to cooperatively bind to the METTL3 active site and increase its activity was initially described by Selberg and colleagues [139] (Fig. 6A, 1 to 4). In the following cellular assays, compound one was the most effective one, which increased the relative m^6^A amount by 21.4 ± 12.9% (Fig. 6B). Lan et al. [140] further presented a photo-activatable small-molecule METTL3 agonist (Fig. 6A, 5 and 6), which fully hid its biological activity by obstructing the functional N–H group on the agonist chemical piperidine-3-carboxylate 1 (MPCH 1). However, after being exposed to the 365-nm light for a short while, its activation was effectively restored, leading to a significant hypermethylation of m^6^A alteration in transcriptome RNAs (Fig. 6C). Dozens of FTO inhibitors have been reported, which can be roughly divided into several types with different structure, binding sites, and binding ability [141]. Given the pro-osteogenic and pro-chondrogenic ability of m^6^A modification, the application of the METTL3 activators or FTO inhibitors in BMSCs seems to offer hope for promoting bone and cartilage formation in vivo. However, the clinical application of these chemical tools is still in its infancy and there is a lack of investigations about their use in BMSCs.

On the contrary, METTL3-selective inhibitors could occupy the SAM binding site of METTL3 and therefore decrease the m^6^A level (Fig. 6D). Using a co-factor mimicking approach, reporting from Yankova et al. identified a selective inhibitor (STM2467) of METTL3 catalytic activity with an IC_50_ (half-maximal inhibitory concentration) of 16.9 nM, and demonstrated its efficacy against myeloid leukemia in vitro and in vivo [142]. STC-15 and UZH1a both serve as METTL3 inhibitors with potential clinical application value in hematological malignancies [143]. Inspired by this, METTL3-selective inhibitor application in BMSCs could be used to treat inflammatory bone diseases.

## m^6^A Modification in Musculoskeletal Disorders

The level of m^6^A modification in the motor system (namely, the musculoskeletal system) is significantly influenced by a plethora of physical and chemical factors in local environments [144,145]. Xu et al. [144] have compiled a review to summarize the METTL3/METTL14 complex’s physiological activities and associated regulation mechanisms in musculoskeletal disorders. However, although there are more than 100 kinds of disorders involving the musculoskeletal system, the investigators only selected four (osteoporosis, rheumatoid arthritis, osteoarthritis, and osteosarcoma) with disparate pathogenesis and regulatory mechanism, and the enrolled studies are mostly basic ones related to METTL3/METTL14.

As shown above, among various kinds of cells in the motor system (e.g., fibroblast, synoviocytes, immune cells, and endothelial cells), the aberrant expression of m^6^A “writers,” “erasers,” and “readers” in BMSCs disrupts bone and cartilage homeostasis in an m^6^A-dependent or m^6^A-independent manner. Based on their role as precursors to osteoblasts, BMSCs are the gold standard for MSC tissue engineering treatment [1,2]. In this section, we sought to shed further light on the expression and function of m^6^A modification in the onset of osteoporosis, osteomyelitis, bone defects, and osteoarthritis, all of which are characterized by the accelerated deterioration of bone or cartilage [144,145]. Special attention was paid to in vivo studies and the possible application of m^6^A-based therapy in BMSCs.

### Osteoporosis

**Fig. 7.**
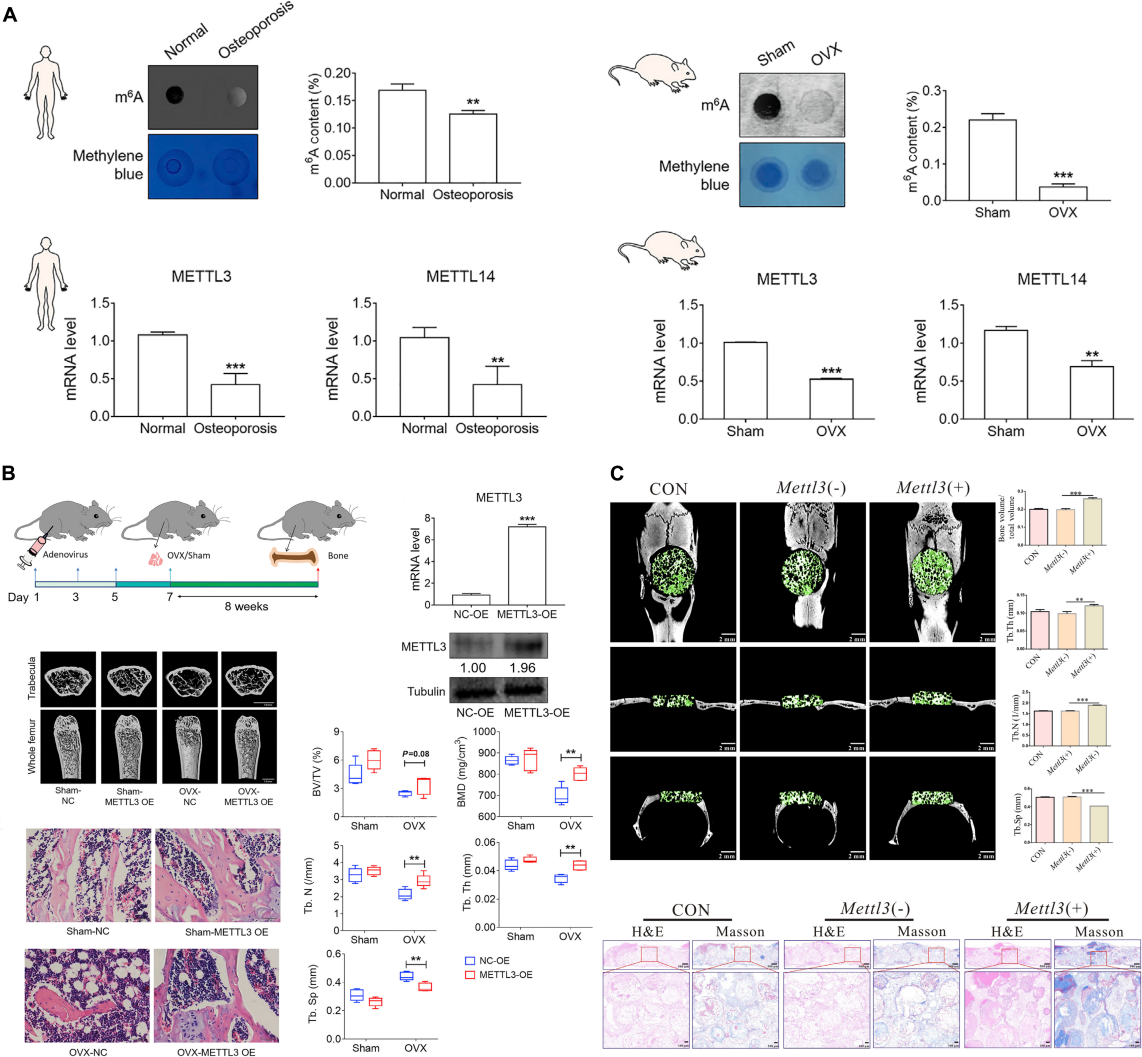
Effect of METTL3 in osteoporosis and bone defect. (A) Global m^6^A level and METTL3/METTL14 expression decrease in osteoporosis bone tissues. Reproduced with permission from [30]. Copyright (2019) the Author(s). (B) Overexpression of METTL3 rescues impaired BMSC function in ovariectomized mice. Reproduced with permission from [30]. Copyright (2019) the Author(s). (C). Overexpression of Mettl3 up-regulated the osteogenic ability of osteoporosis-BMSCs in critical-sized calvarial defects of osteoporosis model rats. Reproduced with permission from [31]. Copyright (2022), the Authors.

Osteoporosis is a chronic systemic bone disease characterized by bone loss, occurring concomitant with an accumulation of bone marrow adipocytes [2]. The BMSCs differentiate preferentially toward adipocytes in response to pathogenic stimuli such as hormone abnormalities or aging, which increases bone loss, fracture risk, and marrow adiposity (Fig. 7A) [30]. Peng et al. [105] collected bone marrow from 32 patients with osteoporosis and found that METTL3 was the most significantly down-regulated “writer” in these patients in comparison with healthy volunteers. Consistent results for METTL3 and METTL14 were obtained in osteoporosis animal models (Fig. 7A) [30,31,146,147]. Decreased methylation levels and lower expression of METTL3/METTL14 were revealed in osteoporosis-BMSCs than in BMSCs from the control group [31,52,54]. Cell fate of BMSCs in mice is disrupted by METTL3 or METTL14 deletion, leading to osteoporosis pathological characteristics including decreased bone mass and accumulated marrow adiposity [49,52,54,146,147]. Consistently, the level of FTO is elevated in BMSCs from patients with osteoporosis and ovariectomy (OVX) mouse [34].

As was already indicated, the overexpression of METTL3 and METTL14 partially restored the osteogenic differentiation of BMSCs by the m^6^A mechanism [30–33,52,54]. Gaining function for METTL3 and METTL14 stops estrogen deficiency-induced postmenopausal osteoporosis [49,54]. These results confirmed that the m^6^A methylation markedly contributes to the maintenance of osteogenesis as a whole, and overexpression of METTL3 and METTL14 in BMSCs has the potential to become a candidate for treating osteoporosis (Fig. 7B). Meanwhile, OVX-induced osteoporosis in mice with ovariectomies was somewhat mitigated by FTO inhibition [34].

### Bone defect

Bone defects occur in many clinical situations such as high-grade open fractures, infection requiring debridement of bone, and resection of bone tumors [148]. For critical-sized bone defects, current treatment options include various natural and synthetic graft materials, such as freeze-dried bone, coral, hydroxylapatite, and tricalcium phosphate [149,150]. In vitro and in vivo investigations have suggested the additional benefits of BMSCs in conjunction with tissue engineering and regenerative medicine in bone repair and regeneration.

The increased osteogenesis of BMSCs by m^6^A modification could also be used in the treatment of bone defects combined with degradable biomaterials. Compared to the control group, BMSCs stimulated by β-tricalcium phosphate (β-TCP) exhibited considerably greater expression of METTL3, which influences m^6^A modification of RNA in BMSCs and improves the stability of RUNX2 mRNA [151]. In animal bone defect models, Jiao et al. [151] discovered that β-TCP increased the m^6^A alteration of RUNX2, which stimulated the production of new bone. Han and colleagues [152] revealed similar results by using mesenchymal stem cells of the apical papillas (SCAPs), odontogenic MSCs with strong osteo/odontogenic capacity. In the nude mouse model transplanted a mixture of SCAPs and HA/tricalcium phosphate as a carrier, miR-196b-5p mimic has favorable effects on the in vivo osteo/odontogenic differentiation of SCAPs in a METTL3-dependent manner [152]. Wu et al. [31] established critical-sized calvarial defects in osteoporosis model rats and implanted biphasic calcium phosphate (BCP) with osteoporosis-BMSCs into the bone defect regions. Eight weeks after transplantation, μ-CT (computed tomography) and H&E (hematoxylin and eosin) staining revealed more bone matrix in the METTL3(+) group than in the METTL3(−) group (Fig. 7C).

### Osteomyelitis

As a typical inflammatory bone disease caused by microorganisms, osteomyelitis can lead to progressive bone necrosis, osteolysis, and bone defect [153]. Although antimicrobial therapy and surgery are the primary treatment strategies, interventions to promote bone formation are also indispensable for chronic or progressive osteomyelitis. Hu and Jiao [154] enrolled 33 osteomyelitis patients and demonstrated up-regulated METTL3 expression in the bone marrow puncture tissue samples in comparison with samples from healthy control. However, METTL3 expressed by osteoblasts was down-regulated in lipopolysaccharide (LPS)-induced inflammation and METTL3 depletion favored proinflammatory cytokine expression of osteoblasts [40]. METTL3 was also closely correlated to immune infiltration and immune response of osteomyelitis [153]. The up-regulated METTL3 level could be explained by the enhanced gene expression in immune cells, especially macrophages.

STM2457 pretreatment down-regulated the expression of MyD88 in bone marrow-derived macrophages and alleviated the symptoms of osteomyelitis in mice [154]. Meanwhile, METTL3 knockdown could inhibit osteoclast differentiation and raise osteoclast apoptosis in inflammatory bone disease by promoting NOS2 mRNA stability in a YTHDF1-dependent manner [155]. Nonetheless, it should be highlighted that the blocking of METTL3, on the one hand, avoids the progression of inflammatory osteolysis and destruction and, on the other hand, impedes the osteogenesis of BMSCs and encourages the survival and proliferation of colonized bacteria [154]. The potential therapeutic benefits of STM2457 for osteomyelitis need a further comprehensive analysis.

### Osteoarthritis

Osteoarthritis is a chronic, degenerative joint disease characterized by the erosion of joint cartilage and inflammation, as well as degradation of the ECM [156,157]. Chondrocyte is the only type of cell found in cartilage, and the activity of chondrocyte is regulated by multiple inflammatory and metabolic factors [158]. Although METTL3 may help the chondrogenesis of BMSCs, the role of this m^6^A “writer” in the development of osteoarthritis was debatable [159,160]. According to data from GSE117999, GSE98918, GSE29746, GSE55457, and GSE82107, translation of METTL3 was down-regulated in cartilage, meniscus, and synovial tissues of patients with osteoarthritis in comparison with the normal control [160,161]. Further experiments enrolling 10 patients with osteoarthritis verified these results by using reverse transcription polymerase chain reaction (RT-PCR) and Western blot [160]. Opposite findings were shown in the experimental collagenase-induced osteoarthritis model constructed by Liu et al. [159], which demonstrated an improved METTL3 mRNA level and percentage of m^6^A methylated mRNA of total mRNA. Two clinical articles also revealed increased expressions of METTL3 mRNA and protein in cartilage of patients with osteoarthritis by using RT-PCR and Western blot [162,163]. However, sample sizes of these two studies are relatively small and whether animal studies could reflect clinical facts remains obscure.

Whether m^6^A hastens or delays the progression of osteoarthritis is also controversial. Mechanistically, m^6^A modification up-regulates the expression of LINC00680 in the osteoarthritis tissue and interleukin-1β (IL-1β)-induced isolated primary chondrocytes, and the latter enhances the mRNA stability of SIRT1, a gene with definite functions in osteoarthritis [163]. Meanwhile, METTL3-mediated m^6^A modification suppresses SOCS2 expression, which activates the JAK2/STAT3 proinflammatory pathway and promotes IL-1β-induced chondrocyte apoptosis, inflammation, and ECM degradation [164]. In contrast, Sang et al.'s [160] study found that METTL3 overexpression decreased the amounts of inflammatory cytokines brought on by IL-1β therapy. From a different angle, m^6^A modification can regulate ECM breakdown in osteoarthritis by balancing the amounts of MMP1, MMP3, MMP13, TIMP-1, and TIMP-2 [157,160,161].

## Conclusion and Future Perspectives

Current studies have revealed that interaction existed between m^6^A modification, mRNAs, miRNAs, lncRNAs, and other ncRNAs, constructing a complicated network and affecting multiple cellular signaling pathways. By modifying RNA metabolism in m^6^A-dependent and m^6^A-independent ways, METTL3 may control the lineage commitment of BMSCs; however, methyltransferase by itself might not be sufficient to determine the direction of differentiation. As a whole, the m^6^A methylation of RNA positively regulates osteogenesis and chondrogenesis of BMSCs, and reverses adipogenesis, mainly achieved by the direct and indirect regulation of specific TFs. These evidences provide the basis for strengthening m^6^A modification in specific musculoskeletal disorders. It should be noted that m^6^A modification is essential for various biological processes, including angiogenesis and bone metabolism [165,166]. To fully comprehend how m^6^A modification affects each and every BMSC osteogenesis signature, more research is required.

As described earlier, osteoporosis and bone defect are characterized by a disruption of bone homeostasis. m^6^A modification encourages bone formation and therefore may prevent the progression of these two musculoskeletal conditions. However, the influence of m^6^A modification on osteomyelitis and osteoarthritis is more convoluted because they are more inflammatory bone diseases besides bone and cartilage destruction. m^6^A modification may aggravate osteomyelitis and osteoarthritis by controlling the activation of pro-inflammatory immune cells, production of inflammatory mediators, and breakdown of the ECM. Clinical application of METTL3 activators/inhibitors (e.g., STM2457, STC-15, and UZH1α) and FTO inhibitors as therapeutic tools for musculoskeletal disorders is still in its infancy. Meanwhile, since the intact catalytic activity of MTC also relies on the function of METTL14 and other binding partners, m^6^A modification inhibitors or activators designed on protein–protein interaction strategy also present reasonable options for regulating lineage commitment of BMSCs.

Nevertheless, current understanding of m^6^A modifications in BMSC differentiation could not be all there is to it. The shortcomings of studies included in this review merit consideration. First, there is controversial evidence about the functions of m^6^A modifications on ncRNAs in the differentiation of BMSCs and the majority of the current evidence was restricted to in vitro confirmation, rarely in clinical value. A more detailed ceRNA network may be constructed to confirm the interaction between ncRNAs and mRNAs. Second, besides the targets we discussed above, there remain other potential mRNAs and ncRNAs that may be involved in the differentiation of BMSCs. Third, bone and cartilage homeostasis is maintained by various cells and ingredients, and therefore, interventions targeting m^6^A modifications in BMSCs alone could not so obviously influence these processes. Last but not least, there are not many clinical investigations on the function of m^6^A modifications in musculoskeletal illnesses.

In this review, we put up novel biological functions and perspectives for the future clinical value of intervening m^6^A modification in BMSCs as a therapeutic regimen for osteoporosis, osteomyelitis, bone defect, and osteoarthritis. Although these four musculoskeletal disorders are accompanied by the destruction of bone and cartilage, m^6^A modification contributes to the course of these diseases in both positive and negative ways. More studies are warranted to further investigate the impact of m^6^A modification on differentiation of BMSCs and verify the efficacy of the m^6^A modification-based therapy.
