# Supplementary figures and images for: N6-Methyladenosine in Cell-Fate Determination of BMSCs: From Mechanism to Applications
Source: Research (Wash D C). 2024 Apr 25;7:0340. doi: 10.34133/research.0340 (PMC11045264; doi:10.34133/research.0340)

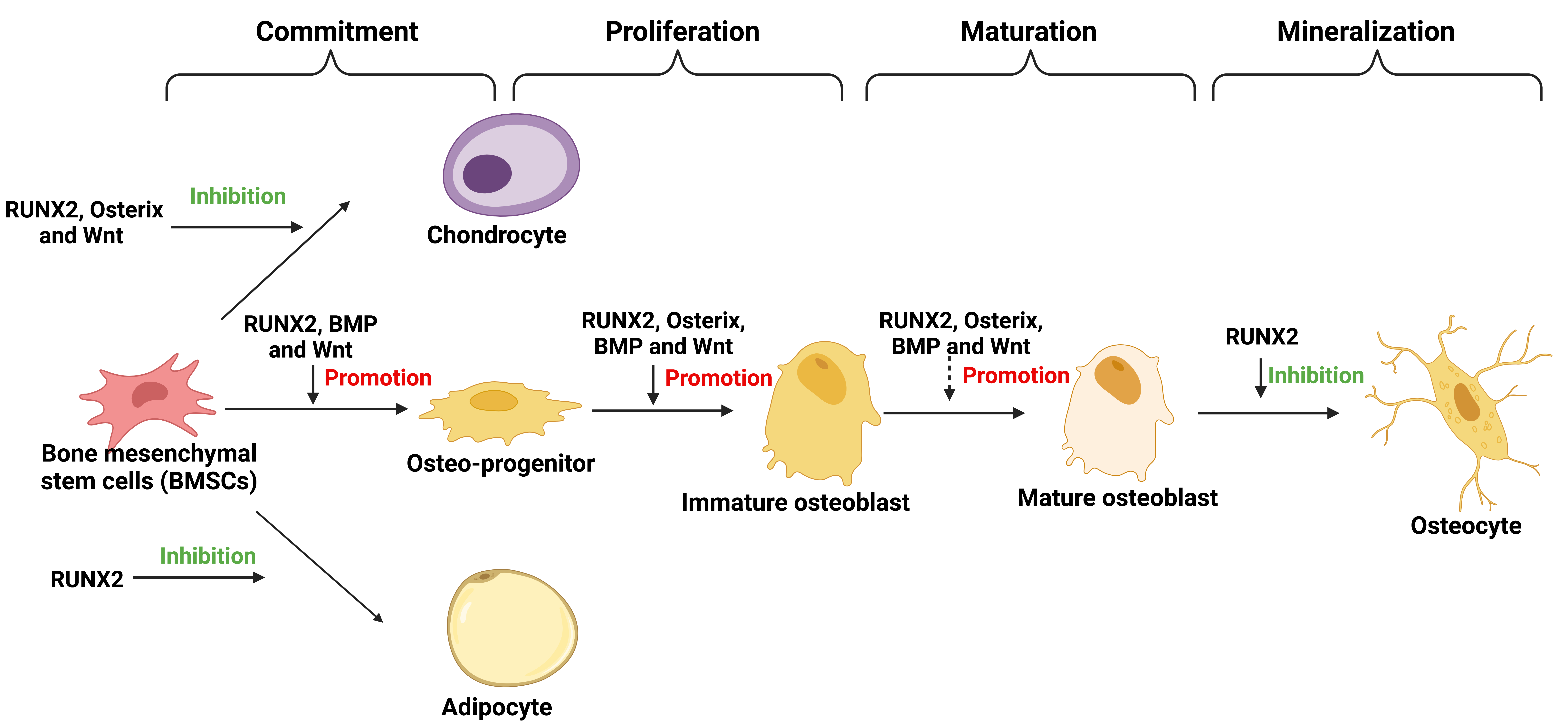

Supplement: Supplementary 1 — Fig. S1 [file research.0340.f1.zip › Supplementary Figure 1.tif]
